# Intelligent Robot Interventions for People With Dementia: Systematic Review and Meta-Analysis of Randomized Controlled Trials

**DOI:** 10.2196/59892

**Published:** 2025-03-10

**Authors:** Wenqi Fan, Rui Zhao, Xiaoxia Liu, Lina Ge

**Affiliations:** 1 Department of Obstetrics and Gynecology, Shengjing Hospital of China Medical University Shenyang China

**Keywords:** intelligent robot, artificial intelligence, dementia, agitation, anxiety, meta-analysis

## Abstract

**Background:**

The application of intelligent robots in therapy is becoming more and more important for people with dementia. More extensive research is still needed to evaluate its impact on behavioral and psychological dementia symptoms, as well as quality of life in different care settings.

**Objective:**

The purpose of this research is to methodically assess how well intelligence robot interventions work for patients with dementia.

**Methods:**

In accordance with the PRISMA (Preferred Reporting Items for Systematic Reviews and Meta-Analyses) 2020 guidelines, a comprehensive search was conducted on PubMed, CINAHL, the Cochrane Library, Embase, and Web of Science from the time of their founding to February 2024, to identify relevant randomized controlled trials on the use of intelligent robots in people with dementia. Two authors (WF and RZ) independently applied the Cochrane Collaboration bias assessment tool to assess the included studies’ quality. The intervention effect of intelligent robots on patients with dementia was summarized using a fixed-effect model or a random-effects model with Stata software (version 16.0; StataCorp). Subgroup analysis was performed according to the intelligent robot type and the intervention duration. Publication bias was tested using funnel plots, Egger tests, and the trim-and-fill method.

**Results:**

In total, 15 studies were finally included for systematic review, encompassing 705 participants, of which 12 studies were subjected to meta-analysis. The meta-analysis found that compared with the control group, intelligent robot intervention significantly reduced the levels of agitation (standardized mean difference –0.36, 95% CI –0.56 to –0.17; *P*<.001) and anxiety (weighted mean difference –1.93, 95% CI –3.13 to –0.72; *P*=.002) in patients with dementia. However, the intervention of intelligent robots had no significant effect on the following (all *P*>.05): cognitive function, neuropsychiatric symptoms, depression, quality of life, step count during the day, and the hours of lying down during the night of patients with dementia. Subgroup analysis revealed that the improvement of depression was related to the duration of the intervention (≤12 vs 12 weeks: 0.08, 95% CI –0.20 to 0.37 vs –0.68, 95% CI –1.00 to –0.37; *P*=.26) and was independent of the type of intelligent robots (animal robots vs humanoid robots: –0.30, 95% CI –0.75 to 0.15 vs 0.07, 95% CI –0.21 to –0.34; *P*=.26).

**Conclusions:**

This study shows that intelligent robot intervention can help improve the agitation and anxiety levels of people with dementia. The intervention may be more effective the longer it is implemented. The appearance of the intelligent robot has no effect on the intervention effect. Further research is needed to help collect physiological data, such as physical activity in people with dementia; explore the impact of other intelligent robot design features on the intervention effect; and provide a reference for improving intelligent robots and intervention programs.

**Trial Registration:**

PROSPERO CRD42024523007; https://tinyurl.com/mwscn985

## Introduction

### Background

As the global aging situation becomes more serious, dementia has become a highly prevalent disease in the older population [1]. The characteristic of dementia is the degradation of memory, cognition, behavior, and daily activity abilities [2]. Symptoms such as agitation and depression increase the risk of secondary problems like fractures and falls, which can seriously impair patients’ quality of life. In addition, it puts more strain on those who provide care, raising the expense of nursing and medical care. According to the August 2020 update of the “Guidelines for Dementia Prevention, Intervention, and Care” by the Lancet Committee, psychotropic medications typically have little effect on neurological or mental symptoms and can have substantial side effects [3]. As a first line of treatment for behavioral and psychological symptoms of dementia, nonpharmacological therapies are advised due to the drawbacks and safety concerns associated with medication therapy [4].

Multiple studies have shown that music therapy and multisensory stimulation intervention have significant effects in improving behavioral and psychological symptoms of dementia [5,6]. The senses enable patients to interact effectively with the environment and communicate better with others. The effects of pet therapy applied in the geriatric population are mainly in the form of improved social interaction, improved emotional state, enhanced cognitive level, and improved problems. Studies on patients with dementia have demonstrated the beneficial effects of pet therapy [7]. Based on the above evidence, using intelligent robots for intervention in patients with dementia may have the following advantages:

1. Multiplicity: Intelligent robots can comprehensively provide tactile, visual, and auditory multisensory stimulation interventions, music interventions, and pet interventions; they can also assist in social activities, accompany patients with dementia, provide social-emotional support, etc.
2. Interactivity: Intelligent robots are capable of interacting with patients, and the interaction modes mainly include touch interaction; voice interaction; and somatosensory interaction, which is a two-way active form rather than one-way passive form and is conducive to improving patient participation and enriching the emotional experience.
3. Telemedicine: The therapist can use remote control software installed in the device to control the activation and progress of the smart robot scripts, enabling telemedicine.
4. Less restriction: The use of intelligent robots to accompany patients is less restricted by place and population compared to animal interventions; for example, patients with hair allergies are not suitable to receive canine companionship but can interact with pet robots.

In recent years, intelligence robot interventions have caught the interest of scientists studying dementia. A variety of intelligent robots, including pet robots, companion robots, social assistance robots, and humanoid robots, are being used for neuropsychiatric symptoms, cognitive function, and pain in patients with dementia [8,9]. The results of intelligence robot intervention vary, nevertheless. For instance, a mixed methods study revealed that following a 6-week intervention, the robotic companion dog or cat group’s feelings of melancholy and loneliness considerably dropped as compared to the control group [10]. The study’s qualitative findings revealed that the robotic companion pet experience was viewed positively by the participants, their families, and professional caregivers, who felt that the robot enhanced communication and offered companionship [10]. Conversely, another study indicated that a social robot intervention did not significantly improve positive affective states [11]. There is currently conflict over the efficacy of interventions on agitation and quality of life, as well as a lack of data for objective indicators like physical activity and sleep duration, despite the publication of a few meta-analyses of intelligence robot intervention [8,9,11-13]. Subgroup analyses of intervention settings, such as the kind and purpose of intelligent robots and the length, the location, and the format of the intervention, have not been done in previous studies. New trials have been published in the last two years; thus, an updated review of this material is still necessary.

### Objectives

Given this, this study aimed to examine how intelligence robots affect both subjective and objective markers in patients with dementia by a thorough assessment of the literature and meta-analysis. Furthermore, the type of intelligent robots, as well as subgroup analysis based on intervention times, are discussed. Overall, the findings will offer support for further relevant studies and therapeutic applications.

## Methods

### Overview

We conducted this systematic review and meta-analysis by following the PRISMA (Preferred Reporting Items in the Systematic Review and Meta-Analyses) guidelines (Multimedia Appendix 1).

### Literature Search Strategy

WF and RZ conducted a comprehensive search in February 2024 across the following databases: Web of Science, CINAHL, PubMed, Embase, Cochrane Library, and CINAHL. We found additional relevant publications by searching the bibliographies of the included papers and previous relevant systematic reviews. Details about the customized search approach for each database can be found in Tables S1-S6 in Multimedia Appendix 2. The following search terms were used: “dementia,” “Alzheimer’s disease,” “AD,” “robotics,” “robotic,” “robot,” and “robot-assisted.”

### Criteria for Inclusion and Exclusion

The included studies satisfied the following criteria: (1) adult patients were diagnosed with dementia; (2) the study was a randomized controlled trial (RCT); (3) the intervention group received intelligence robot intervention, with no restrictions on the kinds of intelligent robots; (4) the control group was given standard treatment; and (5) the study was published in English. The exclusion criteria are as follows: (1) absence of data for the network meta-analysis; (2) duplicate data; and (3) qualitative research, books, review studies, conference abstracts, or study protocols.

### Study Selection and Data Extraction

EndNote 20.4 (Clarivate Analytics) was used to eliminate duplicates before deciding if the searched records were eligible. Initial screening of literature by two independent authors (WF and RZ). WF and RZ discussed and settled any differences between them until they came to an agreement. A third author (XL) was consulted if a consensus could not be achieved. The first author, the country, the year of publication, the type of study, the analyzed sample size, the average age of the participants, the specifics of the intervention (methods, frequency, and duration), and assessment time points were all extracted using a sheet by two independent authors, WF and XL.

### Risk of Bias Assessment

Two authors (WF and RZ) independently applied the Cochrane Collaboration risk of bias tool [14] to assess the quality of the included studies.

### Statistical Analysis

The meta-analysis was conducted using Stata software (version 16.0; StataCorp). To address potential bias caused by differences in indicator levels between the intervention and control groups at baseline, we calculated the mean difference and SD between postintervention and baseline values to reflect within-group changes.

The effect size, which quantifies the difference in the magnitude of change between groups, was expressed as either the standardized mean difference (SMD) or the weighted mean difference (WMD). The WMD was used when studies measured outcomes on the same scale; otherwise, the SMD was applied.

Heterogeneity was considered significant when the *P* value of the Cochran *Q* test was less than 0.10 and the Higgins *I^2^* statistic was greater than 50%, and a random effects model was used to account for heterogeneity between studies; otherwise, a fixed effects model was used [15]. A forest plot and a funnel plot were generated for the analysis. The calculation of Egger regression tests was done to assess publication bias.

Forest plots were generated, and sensitivity analyses were performed to further assess the stability of the study results. The calculation of Egger regression tests, visualization of the funnel plot, and trim-and-fill method were used to evaluate publication bias [16].

Subgroup analyses were conducted to explore the influence of study design factors (intervention duration) and intervention conditions (types of intelligent robots) on the effect of the intervention and to assess possible sources of heterogeneity.

## Results

### Literature Screening

**Figure 1:**
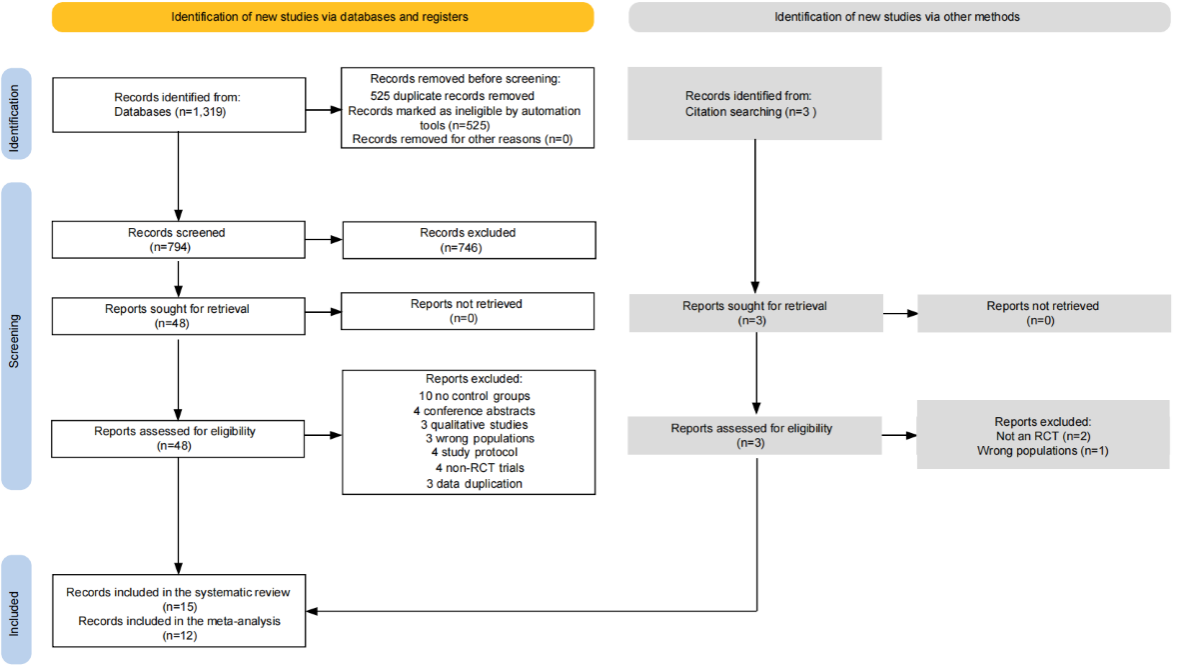
PRISMA flow diagram of the study selection process. PRISMA: Preferred Reporting Items in the Systematic Review and Meta-Analyses; RCT: randomized controlled trial.

The PRISMA flowchart of study identification and screening is outlined in Figure 1. A total of 1319 records were extracted from the electronic database search, and 3 records were identified through hand searching of reference. Following the elimination of duplicates, we went over the abstracts and titles of 797 (60.29%) publications to weed out any that were not relevant. In the end, after verifying compliance with the inclusion criteria and exclusion criteria, a total of 15 (1.13%) studies were included in the systematic review, and 12 (0.91%) of these studies were included in the meta-analysis.

### Study Characteristics

The characteristics of the included studies in the systematic review are shown in Table 1. A total of 15 studies were conducted in 8 countries, including Australia (5/15, 33%) [17-21], Norway (3/15, 20%) [22-24], China (2/15, 13%) [25,26], Spain (1/15, 7%) [27], New Zealand (1/15, 7%) [28], Japan (1/15, 7%) [29], the United Kingdom (1/15, 7%) [30], and the United States (1/15, 7%) [31]. All studies were published after 2015, and the number of participants in each study ranged from 22 to 175, with intervention periods ranging from 6 weeks to 3 months. Participants were mainly female, with the proportion ranging from 64% to 88%, and the average age of participants was reported to be from 83.4 to 89.0 years in 13 (87%) [17-26,29-31] studies. The venues where the interventions were implemented included long-term care facilities, nursing homes, and other venues. Only 2 (13%) [27,29] studies were single-center studies, while the remaining 13 (87%) [17-26,28,30,31] studies were multicenter studies.

**Table 1:** Characteristics of included studies (n=15).

Study (year), country	Study type	Analyzed sample size (IG^a^ and CG^b^)	Women (%)	Age (years)	Intervention methods	Frequency or duration of intervention	Follow-up duration	Assessment time points
Sugiyama and Nakamura [29] (2022), Japan	RCT^c^	14 and 8	86.3%	Mean 89.0 (SD 4.3)	Playing the shiritori game with a dialogue interactive robot	10 minutes/session 3 sessions/week Total: 33 sessions over 11 weeks	12 weeks	T0 T1: 11 weeks
Bradwell et al [30] (2022), United Kingdom	Stratified cluster RCT	26 and 37	77.8%	Mean 87.2 (SD 7.4)	Interacting with pet robots	Total: 516.3 hours over 4 months	4 months	T0 T1: 4 months
Pu et al [17] (2021), Australia; Pu et al [18] (2020), Australia	RCT	21 and 20 21 and 22	70.7% 69.8%	Mean 86.0 (SD 7.5) Mean 86.0 (SD 7.4)	Social robot intervention	30 minutes/session 5 sessions/week Total: 30 sessions over 6 weeks	6 weeks	T0 T1: 1 week T2: 6 weeks T3: After
Jøranson et al [22] (2021), Norway; Jøranson et al [23] (2016), Norway; Jøranson et al [24] (2015), Norway	Cluster RCT	27 and 26	66.7%	Mean 84.6 (SD 6.9)	Intervention for robot-assisted activity	30 minutes/session 2 sessions/week Total: 14 sessions over 12 weeks	12 weeks	T0 T1: 12 weeks
Ke et al [25] (2020), China; Chen et al [26] (2020), China	RCT with an ABAB^d^ withdrawal design	52 and 51	79.6%	Mean 87.2 (SD 7.4)	Social robot intervention	N/Ae over 16 weeks	32 weeks	T0 T1: 8 weeks T2: 16 weeks T3: 24 weeks T4: 32 weeks
Moyle et al [19] (2018), Australia; Mervin et al [20] (2018), Australia; Moyle et al [21] (2017), Australia	Cluster RCT	67 and 53	73.3%	Mean 84.4 (SD 8.0)	Paro robot intervention	15 minutes/session 3 sessions/week Total: 30 sessions over 10 weeks	15 weeks	T0 T1: 5 weeks T2: 10 weeks T3: 15 weeks
Petersen et al [31] (2017), United States	RCT	35 and 26	77%	Mean 83.4 (SD 5.9)	Paro robot intervention	20 minutes/session 3 sessions/week Total: 36 sessions over 12 weeks	3 months	T0 T1: 3 months
Liang et al [28] (2017), New Zealand	RCT	13 and 11	64%	Range 67-98	Paro robot intervention	30 minutes/session 2-3 sessions/week Total: 12-18 sessions over 6 weeks	12 weeks	T0 T1: 6 weeks T2: 12 weeks
Soler et al [27] (2015), Spain	RCT	Phase 1: IG-NAO: 30 ; CG-PARO: 33; CG: 38 Phase 2: IG-PARO: 42; CG: 32	Phase 1: 88% Phase 2: 90%	Phase 1: mean 84.7 (range 68-87) Phase 2: mean 84.7 (range 69-87)	Paro/Nao robot intervention	30-40 minutes/session 2 sessions/week Total: 24 sessions over 12 weeks	12 weeks	T0 T1: 12 weeks

^a^IG: intervention group.

^b^CG: control group.

^c^RCT: randomized controlled trial.

^d^ABAB design, also known as the reversal design, has four phases: a baseline measurement, a measurement under test conditions, a return to the baseline measurement, and a remeasurement under test conditions.

^e^N/A: not applicable.

### Risk of Bias Assessment

Each included RCTs’ bias risk is displayed in Table S7 in Multimedia Appendix 2. A computer-generated random list was used to generate the randomization sequence in 6 of the RCTs [17,19,23,25,27,28,30], while 1 RCT [31] included coin tossing. The risk of selection bias was deemed questionable since the randomization procedure was not reported in detail in 2 other RCTs [28,29]. However, because it was challenging to blind staff members and participants in the intelligent robot interventions, only 1 RCT [19] was deemed to have a low risk of performance bias.

### Analysis of Overall Effects

#### Cognitive Function

**Figure 2:**
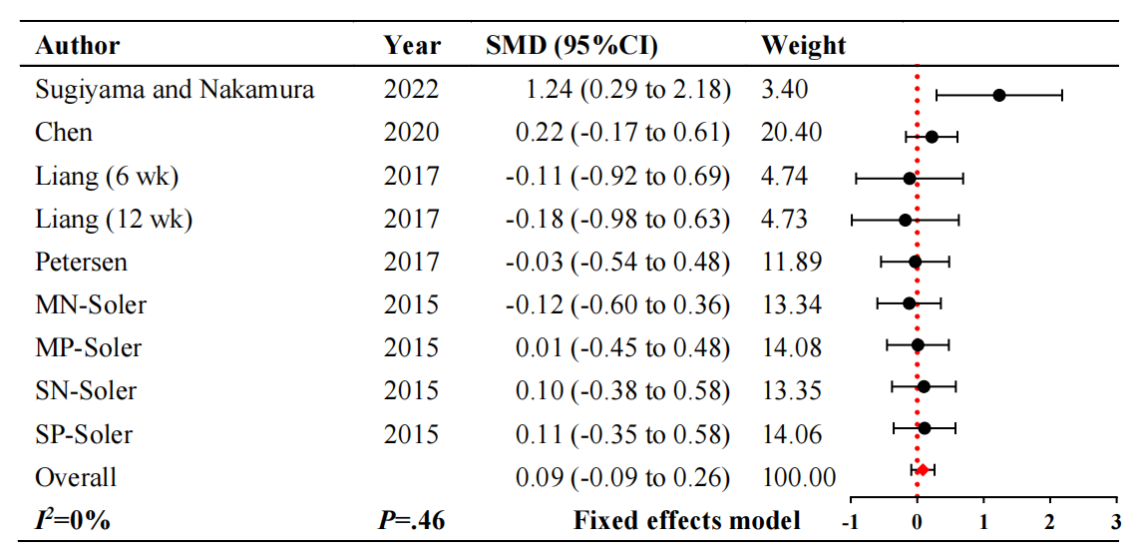
Forest plot of cognitive function. M: Minimental state examination scale; N: Nao robot; P: Paro robot (Pet robot); S: Severe minimental state examination scale. SMD: standardized mean difference.

Cognitive function was measured in 5 studies [26-29,31], and 1 RCT [27] reported humanoid and pet robot intervention with different scales. The combined data showed that there was no discernible variation in cognitive function (SMD 0.09, 95% CI –0.09 to 0.26; *P*=.46). Furthermore, the results showed no heterogeneity (*I²*=0%). In Figure 2 [26-29,31], the forest diagram is displayed.

#### Neuropsychiatric Symptoms

**Figure 3:**
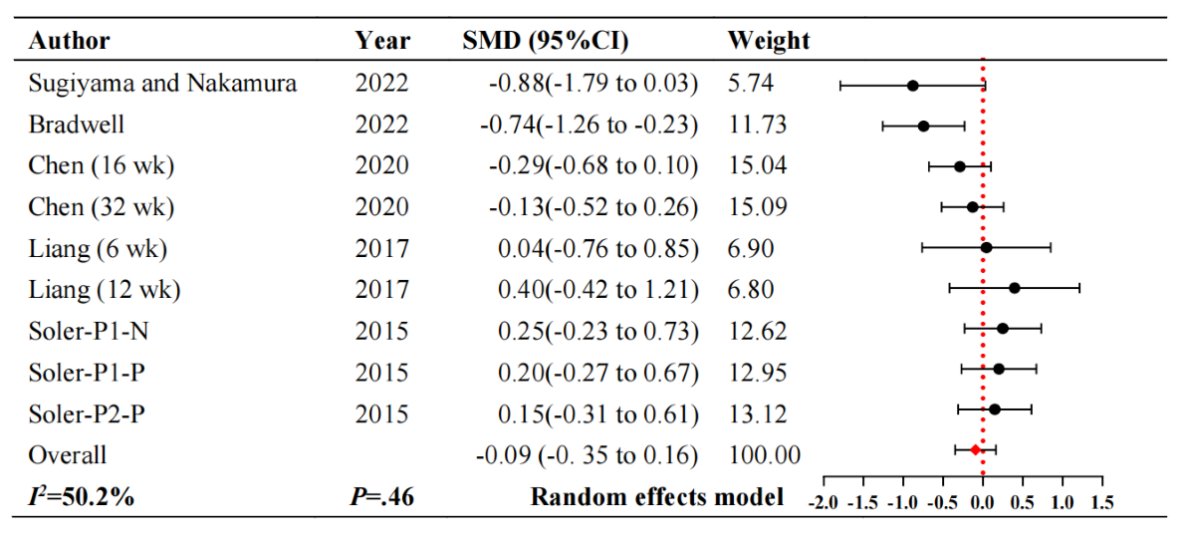
Forest plot of neuropsychiatric symptoms. N: Nao robot (humanoid robot); P: Paro robot (Pet robot); SMD: standardized mean difference.

Regarding neuropsychiatric symptoms, 5 studies [26-30] involving 552 participants reported changes in neuropsychiatric symptoms. Figure 3 [26-30] illustrates that intelligent robot interventions cannot significantly improve neuropsychiatric symptoms in patients with dementia compared to usual care (SMD –0.09, 95% CI –0.35 to 0.16; *P*=.46). These studies showed considerable heterogeneity (*I^2^*=50.2%; *P=*.07).

#### Agitation

**Figure 4:**
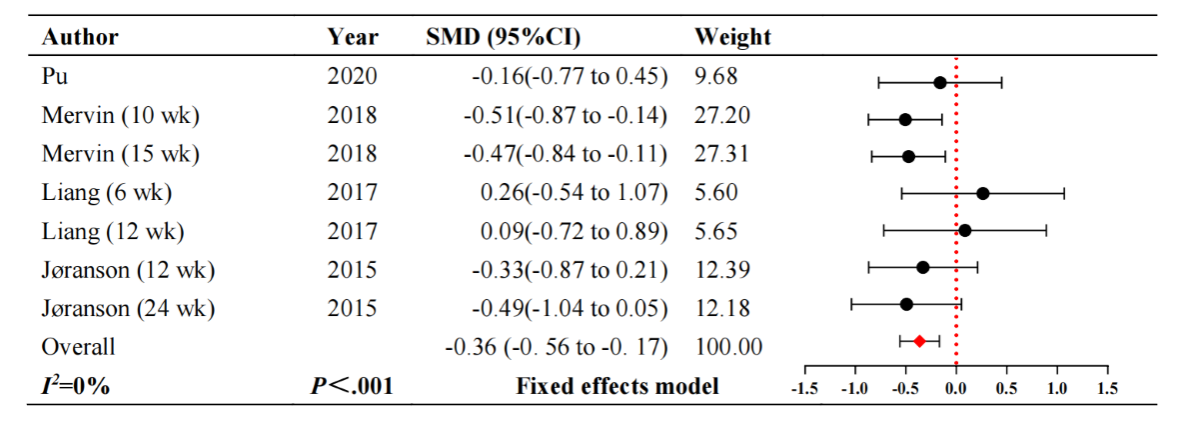
Forest plot of agitation. SMD: standardized mean difference.

Agitation was measured in 4 studies [18,20,24,28]. Figure 4 [18,20,24,28] displays a substantial decline in agitation levels (SMD –0.36, 95% CI –0.56 to –0.17; *P*<.001). These studies showed no heterogeneity (*I^2^*=0).

#### Anxiety

**Figure 5:**
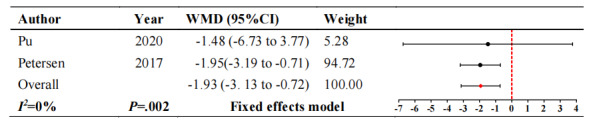
Forest plot of anxiety. WMD: weighted mean difference.

For anxiety, 2 studies [18,31] involving 102 participants reported changes in anxiety. Figure 5 [18,31] illustrates that intelligent robot interventions significantly reduce anxiety scores in patients with dementia compared to usual care (WMD –1.93, 95% CI –3.13 to –0.72; *P*=.002). These studies showed no heterogeneity (*I^2^*=0).

#### Depression

**Figure 6:**
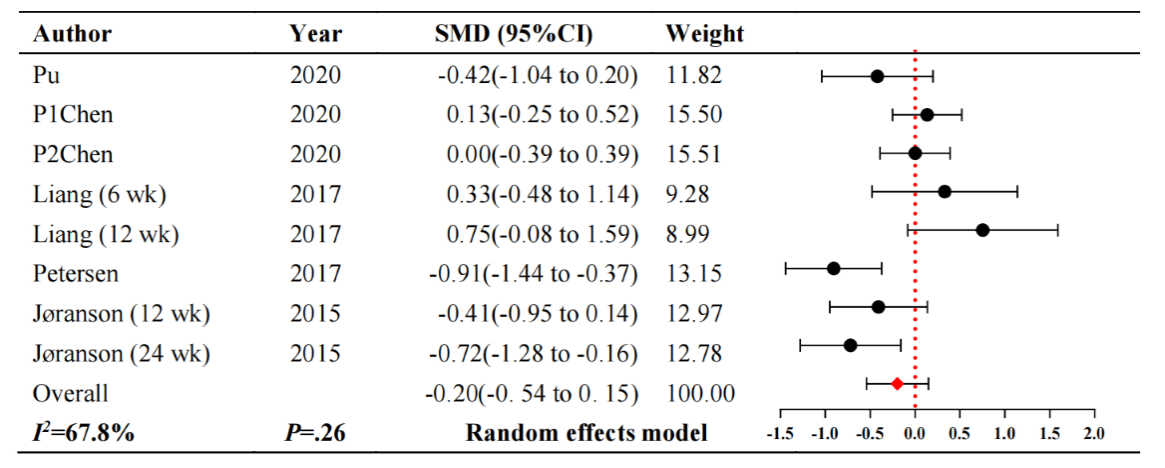
Forest plot of depression. SMD: standardized mean difference.

Regarding depression, 5 studies [18,24,26,28,31] involved 222 participants in the control group and 240 participants in the robot intervention group. Figure 6 [18,24,26,28,31] shows that the intelligent robot interventions cannot relieve depression symptoms in patients with dementia compared to usual care (SMD –0.20, 95% CI –0.54 to 0.15; *P*=.26). These studies showed considerable heterogeneity (*I^2^*=67.8%; *P*=.003).

#### Quality of Life

**Figure 7:**
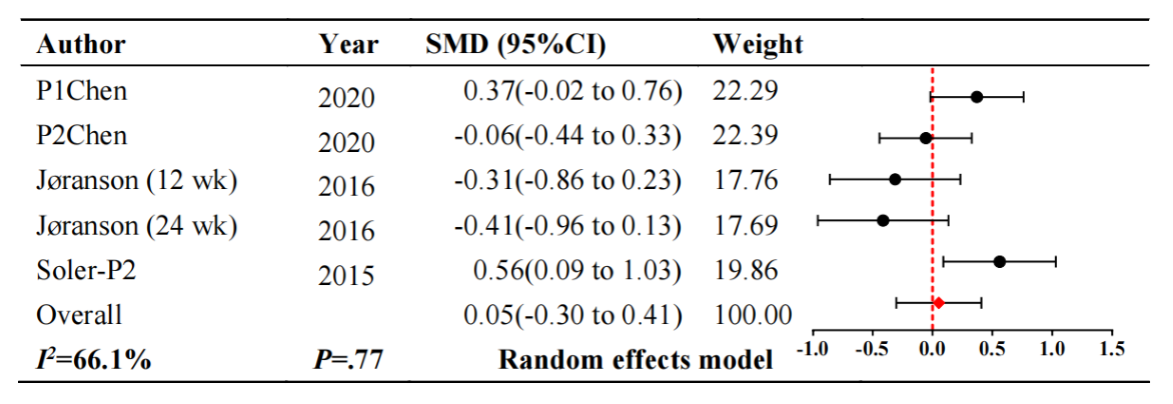
Forest plot of quality of life. SMD: standardized mean difference.

For quality of life, 3 studies [23,26,27] were measured. The quality of life scores displayed in Figure 7 [23,26,27] shows that the intelligent robot interventions cannot improve the quality of life in patients with dementia compared to usual care (SMD 0.05, 95% CI –0.30 to 0.41; *P*=.77). These studies showed considerable heterogeneity (*I^2^*=66.1%; *P*=.02).

#### Data of SenseWear Armband

Sleep and activity data were collected using the SenseWear armband from 2 RCTs [17,19]. The combined data revealed a significant decrease in the amount of time spent lying down during the day (WMD –0.48, 95%CI –0.90 to –0.07; *P*=.02; *I*^2^=0) and reported nonsignificant impacts on length of awake during the daytime or nighttime, length of time lying down during the nighttime, and step count or length of physical activity during the daytime or nighttime (all *P*>.05; Table 2).

**Table 2:** Meta-analysis of sleep and activity data of SenseWear armband.

				WMD^a^	95% CI		*P* value					*I*^2^ (%)
**Daytime**												
	Lying down (hours)		–0.48			–0.90 to –0.07			.02		0.00	
	Awake (hours)		0.22			–0.12 to 0.57			.20		2.10	
	Step count		–31.71			–117.80 to 54.38			.47		43.80	
	Physical activity (hours)^b^		–0.06			–0.36 to 0.24			.69		0.00	
**Nighttime**												
	Lying down (hours)	–0.40			–0.92 to 0.11			.12		0.00		
	Awake (hours)	0.45			–0.07 to 0.97			.09		0.00		
	Step count	–10.54			–45.48 to 24.40			.55		41.90		
	Physical activity (hours)^a^	–0.10			–0.23 to 0.40			.17		0.00		

^a^WMD: weighted mean difference.

^b^Time spent in at least light physical activity (>1.5 metabolic equivalent of task).

### Subgroup Analysis

#### Different Durations of Interventions

##### The Effects on Neuropsychiatric Symptoms

Both short-duration (<12 weeks; SMD –0.18, 95% CI –0.46 to 0.10; *P*=.21; *I*^2^=18%) and long-duration (≥12 weeks; SMD –0.03, 95% CI –0.47 to 0.42; *P*=.91; *I*^2^=70.3%) intelligent robot interventions were not found to enhance neuropsychiatric symptoms significantly (Figure S1 in Multimedia Appendix 2).

##### The Effects on Agitation

The subgroup analysis results showed that different intervention durations significantly improved agitation in people with dementia. Still, the longer the intervention continued, the better the effect (12 weeks; SMD –0.41, 95% CI –0.80 to –0.03; *P*=.04; *I*^2^=0%; Figure S2 in Multimedia Appendix 2).

##### The Effects on Depression

A significant effect favoring the more extended intervention on relieving depression (12 weeks; SMD –0.68, 95% CI –1.00 to –0.37; *P*<.001; *I*²=0; Figure S3 in Multimedia Appendix 2).

##### The Effects on Quality of Life

There was no significant difference between different duration intelligent robot interventions and usual care in improving quality (Figure S4 in Multimedia Appendix 2).

#### Different Types of Intelligent Robots

There was no significant difference between different types of intelligent robot interventions and usual care in improving cognitive function, neuropsychiatric symptoms, depression, and quality of life (Figures S5-S8 in Multimedia Appendix 2).

### Sensitivity Analysis

The research findings of the five outcome measures, namely cognitive function, neuropsychiatric symptoms, agitation, depression, and quality of life, indicate that the point estimates of the combined effect sizes were obtained after removing 1 study at a time and analyzing the remaining studies. The results showed that the exclusion of a particular study does not significantly alter the overall results, indicating that an evaluation of the 5 outcomes had considerable stability (Figures S9-S13 in Multimedia Appendix 2).

### Publication Bias

Funnel plots and Egger tests were performed to evaluate publication bias of 5 outcome measures, namely cognitive function, neuropsychiatric symptoms, agitation, depression, and quality of life. The results showed no publication bias in cognitive function, neuropsychiatric symptoms, depression, and quality of life (Figures S14-S17 in Multimedia Appendix 2). For agitation, the results of the Egger test showed possible publication bias. There was no significant difference between the effect size estimates obtained using the trim-and-fill method and those obtained by meta-analysis, indicating the results were reliable (Figures S18 and S19 in Multimedia Appendix 2).

### Description of Outcomes Not Suitable for Meta-Analysis

A total of 3 studies [21-22,25] were left out of the meta-analysis due to the inability to combine the inclusion of several datasets. Jøranson et al [22] looked into how the intelligent robot Paro affected the sleep habits of patients with dementia living in a nursing home. This study used wrist actigraphy to assess objectively sleep-wake patterns and showed that an intelligent robotic intervention significantly increased the percentage of sleep efficiency and reduced the frequency of nocturnal awakenings in patients with Alzheimer disease. Ke et al [25] assessed the effects of the humanoid social robot (Kabochan) on technology acceptance among older individuals with dementia; they found that being exposed to intelligent robots may enhance their perceptions of the technology’s utility and attitudes toward it. Moyle et al [21] showed that participants in the PARO group were more verbally and visually engaged than participants in plush toy.

## Discussion

### Principal Findings

This study indicates that intelligence robot interventions effectively influenced agitation, anxiety, and length of time lying down during the daytime. The current meta-analysis did discover, however, that intelligence robot interventions had no discernible effects on depression, quality of life, cognitive function, neuropsychiatric symptoms, sleep duration, wakefulness during the day or night, step count, or frequency of physical activity.

In this study, intelligent robot intervention significantly reduces levels of anxiety in patients with dementia, which is consistent with meta-analytic reviews of Saragih et al [8]. Anxiety is highly prevalent across dementia stages, with an overall pooled prevalence of 39% [32]. In recent years, various clinical applications of intelligent robots have been used to provide high-quality emotional support and companionship [33]. Intelligent robots can provide emotional support and companionship to patients with dementia, easing anxiety by interacting with them and sharing stories, music, or simple games. In future studies, the effects of different design elements in intelligent robot interventions on the anxiety levels of patients with dementia can be further explored to understand the preferences of patients with dementia for the appearance characteristics and interaction contents of intelligent robots, so as to improve the references for the design of intelligent robots and clinical application programs, and to give full play to their effectiveness in alleviating the anxiety levels of patients with dementia.

Agitation improved in the intelligent robot intervention group. One study has shown that providing patients with dementia with sensory stimulation, mainly through sight, sound, touch, taste, and smell, can help to improve agitation [34]. Intelligent robots integrate the application of visual, tactile, and auditory multiple sensing technologies, such as the social robot (Paro) covered with artificial fur, capable of moving and emitting sounds to achieve multisensory stimulation, which can put patients with dementia in a multisensory enriched environment and alleviate their agitated state. It has been shown that agitation prolongs the hospital stay of people with dementia and imposes physical, psychological, and financial burdens on caregivers [35]. More research is needed in the future to evaluate whether these interventions also benefit shorter hospital stays, better caregiver psychological health, and lower costs.

The intelligent robot intervention was not significant for overall depression levels in patients with dementia, which is consistent with the findings of Abbott et al [36]. Subgroup analyses based on the length of the intervention were carried out, and the findings indicated that when the intervention lasted for 12 weeks, the intelligent robot intervention helped patients with dementia experience lower levels of depression; however, no such beneficial effect was observed when the intervention lasted for shorter than 12 weeks. Depression in people with dementia often involves deeper emotional issues, and the provision of emotional support and cognitive stimulation by intelligent robots may take longer to produce significant improvements [12]. Subsequent investigations ought to delve deeper into the impact of intervention duration on the efficacy of intelligent robot interventions. The humanoid and animal robots did not differ from one another in subgroup analyses depending on the type of intelligent robot, and neither had a significant impact on the depression levels of patients with dementia. The usefulness of humanoid and animal robot treatments has not been the subject of as many studies, and this study’s sample size was limited. In order to provide some guidance for the appearance design and function setting of intelligent robots, the sample size should be increased in subsequent research, and various types of intelligent robots should be thoroughly examined.

Cognitive function and neuropsychiatric symptoms in patients with dementia did not significantly improve with intelligent robot interventions. The cognitive deterioration involved in dementia is usually associated with structural changes in the brain [37], and although intelligent robots can provide social interaction and behavioral stimulation, these may not be sufficient to reverse or significantly improve the cognitive impairment caused by neurodegenerative diseases. Intelligent robots lack the human caregiver’s capacity for emotional depth, empathy, and complex decision processing, and their role in neuropsychiatric symptoms in patients with dementia may be limited.

The relevant data for this study came from wearable devices, and the included studies reported difficulties in wearing wearable devices in patients with dementia. The results did not suggest a significant positive effect on sleep and physical activity. Given that individuals with dementia were reticent to wear armbands, particularly at night [17], this conclusion should be interpreted cautiously. To increase patient happiness and participation, more appropriate techniques for tracking sleep and physical activity in patients with dementia should be investigated in the future.

In the group receiving intervention, the quality of life did not improve. Studies have shown that one of the main factors influencing the quality of life of patients with dementia is their capacity to carry out activities of daily living [38]. Patients with dementia also tend to be less capable of taking care of themselves, and some even experience difficulties walking. Although intelligent robots can provide a certain degree of life guidance and support, they cannot completely replace human care and assistance. The cognitive abilities, neuropsychiatric symptoms, the stress of being the primary caregiver, and other factors all affect the quality of life of patients with dementia [39].

Subgroup analysis according to the length of intervention showed that intelligent robot intervention for 12 weeks or more had a more positive effect on the depression level and agitation of patients with dementia, and there was no significant difference in neuropsychiatric symptoms and quality of life. In patients with dementia receiving 12 weeks or more of intervention, symptoms such as depression and agitation may change more significantly due to improvements in emotional regulation and behavioral interventions. However, neuropsychiatric symptoms and quality of life are affected by a combination of more complex physical, psychological, and social factors, and the effect of the intervention may not be significantly different in these dimensions. This suggests that although long-term interventions have a positive impact on mood and behavior, longer or more comprehensive interventions may be needed to improve neurodegenerative lesions and quality of life. A subgroup analysis was performed according to whether the intelligent robot was a humanoid robot or a pet robot. The results showed that there was no significant difference in the improvement of cognitive function, neuropsychiatric symptoms, depression, and quality of life in patients with dementia between the two types of robots. Although humanoid robots and pet robots differ in appearance and behavior, their core role in providing intervention is often similar, that is, to improve the mood and behavior of patients through interaction, emotional companionship, cognitive training, and other means. This may be the reason for the similar intervention effects of the two.

### Strengths and Limitations

This study has several practical implications for practice. First, interventions based on intelligent robots can be considered for inclusion in the long-term care of people with dementia. Second, the shape characteristics of intelligent robots may have no effect on the intervention effect on people with dementia, and other design characteristics need to be further explored. Third, when using wearable devices to assess sleep, physical activity, etc, in people with dementia, the wearing comfort and compliance of people with dementia should be considered.

The following limitations of this study need to be taken into account when interpreting the results. First, the sample size included in this study was small, and further analysis of the impact of factors such as the age, gender, and geographical location of participants on the effectiveness of the intervention was lacking. Second, only original studies published in English were included, and there is a possibility that relevant studies published in other languages were missed. In addition, not all studies were assessed by professional blinders, which may introduce bias into the study.

Despite these limitations, this review aims to comprehensively evaluate the impact of intelligent robot interventions on people with dementia. Seven outcome indicators, including objective indicators of sleep and activity, were meta-analyzed. The impact of the intervention duration and robot type on smart robot interventions was further explored to provide a reference for the future optimization of smart robot development and intervention program design.

### Conclusions

This meta-analysis comprehensively assessed the impact of intelligent robot interventions on people with dementia and showed that intelligent robot interventions can reduce agitation and anxiety, with a limited role in improving depression, anxiety, cognitive functioning, neuropsychiatric symptoms, and quality of life, and that sleep- and physical activity-related outcomes need to be analyzed with caution. Subgroup analyses showed better results for the longer the duration of the intelligent robot intervention. The types of intelligent robots included humanoid robots and animal robots, and subgroup analyses showed no difference in their effectiveness. The use of intelligent robots in nursing is still in its infancy, although a number of nations have implemented intelligent robotic interventions for patients with dementia. These interventions have primarily involved women and have small sample sizes and have been concentrated in high-income nations, which may be related to the epidemiological features of dementia. Subsequent research endeavors ought to enhance the conceptual framework of sentient robots and delve deeper into the ways in which the nature of the sentient robot and the length of the intervention affect its efficacy. When using wearable technology to gather physiological data, researchers should take patient compliance into account.

## Appendix

Multimedia Appendix 1 PRISMA (Preferred Reporting Items for Systematic Reviews and Meta-Analyses) 2020 checklist.

Multimedia Appendix 2 Supplementary tables and figures.

## Abbreviations

PRISMA: Preferred Reporting Items for Systematic Reviews and Meta-Analyses
RCT: randomized controlled trial
SMD: standardized mean difference
WMD: weighted mean difference

## Data Availability

The datasets generated or analyzed during this study are available from the corresponding author upon reasonable request.
