# Supplementary material for: Intelligent Robot Interventions for People With Dementia: Systematic Review and Meta-Analysis of Randomized Controlled Trials
Source: J Med Internet Res. 2025 Mar 10;27:e59892. doi: 10.2196/59892 (PMC11933762; doi:10.2196/59892)
Supplement: Multimedia Appendix 2 [file jmir_v27i1e59892_app2.doc]

**Table S1** Search terms.

|  | Mseh terms | Free-text terms |
| --- | --- | --- |
| Population | Dementia  Alzheimer Disease | Dementia  AD  Alzheimer Disease |
| Intervention | Robotics | Robotic  Robot  Robot-Assisted |

**Table S2.** Literature search method of PubMed.

| PubMed | | |
| --- | --- | --- |
| item | Search strategies | n |
| #1 | "Dementia"[MeSH Terms] OR "alzheimer disease"[MeSH Terms] OR "Dementia"[Title/Abstract] OR "AD"[Title/Abstract] OR "alzheimer syndrome"[Title/Abstract] | 382,222 |
| #2 | "robotics"[MeSH Terms] OR "Robotic"[Title/Abstract] OR "Robot"[Title/Abstract] OR "Robot-Assisted"[Title/Abstract] | 73,573 |
| #3 | #1 AND #2 | 387 |

**Table S3.** Literature search method of Cochrane Library.

| Cochrane Library | | |
| --- | --- | --- |
| item | Search strategies | n |
| #1 | MeSH descriptor: [Dementia] this term only  OR MeSH descriptor: [Alzheimer Disease] this term only  OR (Dementia):ti,ab,kw OR (AD):ti,ab,kw  OR (Alzheimer Syndrome):ti,ab,kw | 39,810 |
| #2 | MeSH descriptor: [robotics] this term only  OR MeSH descriptor: [Robotic] this term only  OR (Robot):ti,ab,kw OR (Robotic):ti,ab,kw  OR (Robot-Assisted):ti,ab,kw | 7,193 |
| #3 | #1 AND #2 | 116 |

**Table S4.** Literature search method of EMBASE.

| EMBASE | | |
| --- | --- | --- |
| item | Search strategies | n |
| #1 | 'dementia'/exp OR 'alzheimer disease'/exp OR dementia:ti,ab,kw OR ad:ti,ab,kw OR 'alzheimer syndrome':ti,ab,kw | 641,152 |
| #2 | 'robot'/exp OR 'robotics'/exp OR robotic:ti,ab,kw OR robot:ti,ab,kw OR 'robot assisted':ti,ab,kw | 115,876 |
| #3 | #1 AND #2 | 773 |
| #4 | #3 AND 'article'/it | 381 |

**Table S5.** Literature search method of Web of Science.

| Web of Science | | |
| --- | --- | --- |
| item | Search strategies | n |
| #1 | (Title) OR Alzheimer Disease (Title) OR AD (Title) OR Alzheimer Syndrome (Topic) | 224,798 |
| #2 | (Title) OR Robot (Title) OR Robot-Assisted (Title) | 220,740 |
| #3 | #1 AND #2 | 324 |

**Table S6.** Literature search method of CINAHL.

| CINAHL | | |
| --- | --- | --- |
| item | Search strategies | n |
| #1 | SU Dementia OR SU Alzheimer Disease OR SU AD OR SU Alzheimer Syndrome | 53,957 |
| #2 | SU Robotic OR SU Robot OR SU Robot-Assisted | 8,878 |
| #3 | #1 AND #2 | 111 |

Table S7. Risk of bias summary.[17,19,23,25,27-31]

| Study | a | b | c | d | e | f | g |
| --- | --- | --- | --- | --- | --- | --- | --- |
| Sugiyama and Nakamura (2022)[29] | unclear | unclear | unclear | unclear | low | low | low |
| Bradwell et al (2022)[30] | low | low | unclear | unclear | low | low | high |
| Pu et al (2021)[17] | low | low | high | high | low | low | low |
| Jøranson et al (2016)[23] | low | low | high | high | low | low | high |
| Ke et al (2020)[25] | low | low | unclear | unclear | low | low | low |
| Moyle et al (2018)[19] | low | low | low | unclear | low | low | high |
| Petersen et al (2017)[31] | low | unclear | unclear | unclear | low | low | low |
| Liang et al (2017)[28] | unclear | unclear | unclear | unclear | low | low | low |
| Soler et al (2015)[27] | low | low | unclear | low | low | low | low |
| % with high or unclear risk | 20% | 30% | 90% | 80% | 0% | 0% | 40% |
| a=random sequence generation (selection bias): b= allocation concealment (selection bias): c=Blinding of personnel (nerformance bias): d=blinding of outcome assessment (detection bias): e=incompleteoutcome data (attrition bias); f= Selective reporting (reporting bias); g=other bias | | | | | | | |

**Figure S1.** Subgroup analysis of durations of interventions on neuropsychiatric symptoms.[26-30]

**Figure S2.** Subgroup analysis of durations of interventions on agitation.[18,20,24,28]

**Figure S3.** Subgroup analysis of durations of interventions on depression.[18,24,26,28,31]

**Figure S4.** Subgroup analysis of durations of interventions on quality of life.[23,26,27]

**Figure S5.** Subgroup analysis of robot types on cognitive function.[26-29,31]

**Figure S6.** Subgroup analysis of robot types on neuropsychiatric symptoms.[26-30]

**Figure S7.** Subgroup analysis of robot types on depression.[18,24,26,28,31]

**Figure S8.** Subgroup analysis of robot types on quality of life.[23,26,27]

**Figure S9.** The sensitivity analysis chart for cognitive function.[26-29,31]

**Figure S10.** The sensitivity analysis chart for neuropsychiatric symptoms.[26-30]

**Figure S11.** The sensitivity analysis chart for agitation.[18,20,24,28]

**Figure S12.** The sensitivity analysis chart for depression.[18,24,26,28,31]

**Figure S13.** The sensitivity analysis chart for quality of life.[23,26,27]

**Figure S14.** Funnel plot of cognitive function.[26-29,31]

Egger tests of cognitive function (*P* = 0.52＞0.05 ).

**Figure S15.** Funnel plot of neuropsychiatric symptoms.[26-30]

Egger tests of neuropsychiatric symptoms (*P* = 0.90＞0.05 ).

**Figure S16.** Funnel plot of depression.[18,24,26,28,31]

Egger tests of depression (*P* = 0.98＞0.05 ).

**Figure S17.** Funnel plot of quality of life.[23,26,27]

Egger tests of quality of life (*P* = 0.39＞0.05 ).

**Figure S18.** Funnel plot of agitation.[18,20,24,28]

Egger tests of agitation (*P* = 0.005＜0.05 ).

**Figure S19.** Filled funnel plot of agitation.[18,20,24,28]

**References**

17. Pu L, Moyle W, Jones C, Todorovic M. The effect of a social robot intervention on sleep and motor activity of people living with dementia and chronic pain: a pilot randomized controlled trial. Maturitas. Feb 2021;144:16-22. [doi: 10.1016/j.maturitas.2020.09.003] [Medline: 33358203]

18. Pu L, Moyle W, Jones C, Todorovic M. The effect of using PARO for people living with dementia and chronic pain: a pilot randomized controlled trial. J Am Med Dir Assoc. Aug 2020;21(8):1079-1085. [doi: 10.1016/j.jamda.2020.01.014] [Medline: 32122797]

19. Moyle W, Jones C, Murfield J, Thalib L, Beattie E, Shum D, et al. Effect of a robotic seal on the motor activity and sleep patterns of older people with dementia, as measured by wearable technology: a cluster-randomised controlled trial. Maturitas. Apr 2018;110:10-17. [FREE Full text] [doi: 10.1016/j.maturitas.2018.01.007] [Medline: 29563027]

20. Mervin MC, Moyle W, Jones C, Murfield J, Draper B, Beattie E, et al. The cost-effectiveness of using PARO, a therapeutic robotic seal, to reduce agitation and medication use in dementia: findings from a cluster-randomized controlled trial. J Am Med Dir Assoc. Jul 2018;19(7):619-622.e1. [doi: 10.1016/j.jamda.2017.10.008] [Medline: 29325922]

21. Moyle W, Jones CJ, Murfield JE, Thalib L, Beattie ERA, Shum DKH, et al. Use of a robotic seal as a therapeutic tool to improve dementia symptoms: a cluster-randomized controlled trial. J Am Med Dir Assoc. Sep 01, 2017;18(9):766-773. [FREE Full text] [doi: 10.1016/j.jamda.2017.03.018] [Medline: 28780395]

22. Jøranson N, Olsen C, Calogiuri G, Ihlebæk C, Pedersen I. Effects on sleep from group activity with a robotic seal for nursing home residents with dementia: a cluster randomized controlled trial. Int Psychogeriatr. Oct 2021;33(10):1045-1056. [FREE Full text] [doi: 10.1017/S1041610220001787] [Medline: 32985396]

23. Jøranson N, Pedersen I, Rokstad AMM, Ihlebaek C. Change in quality of life in older people with dementia participating in Paro-activity: a cluster-randomized controlled trial. J Adv Nurs. Dec 2016;72(12):3020-3033. [doi: 10.1111/jan.13076] [Medline: 27434512]

24. Jøranson N, Pedersen I, Rokstad AMM, Ihlebæk C. Effects on symptoms of agitation and depression in persons with dementia participating in robot-assisted activity: a cluster-randomized controlled trial. J Am Med Dir Assoc. Oct 01, 2015;16(10):867-873. [doi:10.1016/j.jamda.2015.05.002] [Medline: 26096582]

25. Ke C, Lou VW, Tan KC, Wai MY, Chan LL. Changes in technology acceptance among older people with dementia: the role of social robot engagement. Int J Med Inform. Sep2020;141:104241-104249. [doi: 10.1016/j.ijmedinf.2020.104241] [Medline: 32739611]

26. Chen K, Lou VW, Tan KC, Wai MY, Chan LL. Effects of a humanoid companion robot on dementia symptoms and caregiver distress for residents in long-term care. J Am Med Dir Assoc. Nov 2020;21(11):1724-1728.e3. [doi: 10.1016/j.jamda.2020.05.036] [Medline: 32713772]

27. Soler MV, Agüera-Ortiz L, Rodríguez JO, Rebolledo CM, Muñoz AP, Pérez IR, et al. Social robots in advanced dementia. Front Aging Neurosci. 2015;7:133-144. [FREE Full text] [doi:10.3389/fnagi.2015.00133] [Medline: 26388764]

28. Liang A, Piroth I, Robinson H, MacDonald B, Fisher M, Nater UM, et al. A pilot randomized trial of a companion robot for people with dementia living in the community. J Am Med Dir Assoc. 2017;18(10):871-878. [doi: 10.1016/j.jamda.2017.05.019] [Medline: 28668664]

29. Sugiyama H, Nakamura K. Temporary improvement of cognitive and behavioral scales for dementia elderly by shiritori word game with a dialogue robot: a pilot study. Front Robot AI. 2022;9:941056-941072. [FREE Full text] [doi: 10.3389/frobt.2022.941056] [Medline: 36530499]

30. Bradwell H, Edwards KJ, Winnington R, Thill S, Allgar V, Jones RB. Implementing affordable socially assistive pet robots in care homes before and during the COVID-19 pandemic: stratified cluster randomized controlled trial and mixed methods study. JMIR Aging. Aug 24, 2022;5(3):e38864. [FREE Full text] [doi: 10.2196/38864] [Medline: 35830959]

31. Petersen S, Houston S, Qin H, Tague C, Studley J. The utilization of robotic pets in dementia care. J Alzheimers Dis. 2017;55(2):569-574. [FREE Full text] [doi: 10.3233/JAD-160703] [Medline: 27716673]
